# hGBP-1 Expression Predicts Shorter Progression-Free Survival in Ovarian Cancers, While Contributing to Paclitaxel Resistance

**DOI:** 10.4236/jct.2016.713097

**Published:** 2016-12-08

**Authors:** Suzan Wadi, Aaron R. Tipton, Jill A. Trendel, Sadik A. Khuder, Deborah J. Vestal

**Affiliations:** 1 Department of Biological Sciences, University of Toledo, Toledo, OH, USA; 2 Department of Medicine, University of Toledo, Toledo, OH, USA

**Keywords:** Guanylate-Binding Protein, Paclitaxel, Ovarian Cancer, Drug Resistance, GTPase, TUBB3

## Abstract

Ovarian cancer is the gynecological cancer with the poorest prognosis. One significant reason is the development of resistance to the chemotherapeutic drugs used in its treatment. The large GTPase, hGBP-1, has been implicated in paclitaxel resistance in ovarian cell lines. Forced expression of hGBP-1 in SKOV3 ovarian cancer cells protects them from paclitaxel-induced cell death. However, prior to this study, nothing was known about whether hGBP-1 was expressed in ovarian tumors and whether its expression correlated with paclitaxel resistance. hGBP-1 is expressed in 17% of ovarian tumors from patients that have not yet received treatment. However, at least 80% of the ovarian tumors that recurred after therapies that included a tax-ane, either paclitaxel or docetaxel, were positive for hGBP-1. In addition, hGBP-1 expression predicts a significantly shorter progression-free survival in ovarian cancers. Based on these studies, hGBP-1 could prove to be a potential biomarker for paclitaxel resistance in ovarian cancer.

## 1. Introduction

Ovarian cancer is the most deadly of the gynecologic cancers. Resistance to chemothe-rapeutics, both innate and acquired, contributes to this poor prognosis. As many as 20% – 25% of patients have innate drug resistance and fail to respond to chemotherapy initially [1]. Although 75% of ovarian tumors will initially respond to chemotherapy, development of drug resistance and tumor recurrence are frequent [1]. Treatment usually involves a drug cocktail containing a taxane, most frequently paclitaxel. This makes the development of resistance to paclitaxel a significant problem in the treatment of ovarian cancer.

To identify a gene signature for paclitaxel resistance, investigators made three cancer cell lines resistant to paclitaxel [2]. Only eight genes were up-regulated in common when all three cell lines became resistant. One of these was the large GTPase, human Guanylate-Binding Protein-1 (hGBP-1) [3]. The Guanylate-Binding Proteins (GBPs) are a family of large, cytokine-induced GTPases (reviewed in [4]). hGBP-1 can be expressed in several types of primary tumors and the tumor-associated cells within them. Depending on the tumor type, the prognosis associated with hGBP-1 expression differs [5] [6] [7] [8] [9]. Forced over-expression of hGBP-1 in paclitaxel-sensitive OVCAR8 ovarian cancer cells resulted in a four-fold increase in IC_50_ for paclitaxel [3]. While not identified in the gene signature described above, TUBB3 has been implicated in paclitaxel resistance and been suggested to interact with and be regulated by hGBP-1 in cultured ovarian cancer cell lines.

We are the first to examine hGBP-1 expression in ovarian tumor samples. We find that hGBP-1 is expressed in only 17% of newly diagnosed ovarian cancers prior to treatment but in at least 80% of ovarian tumors that recur after treatments that include a taxane. The expression of hGBP-1 predicts shorter progression-free survival (PFS) in ovarian cancers of all stages, histologies, and grades provided that they received optimal debulking. These patients subsequently received chemotherapy that included both paclitaxel and platinum [10]. Based on this and other studies, hGBP-1 may be an attractive biomarker for predicting prognosis. In addition, it may be a therapeutic target in ovarian cancers once we learn how hGBP-1 protects cells from chemotherapeutic drugs.

## 2. Materials and Methods

### 2.1. Cells and Plasmids

Cells were obtained from American Type Culture Collection [11]. To generate pCMV_2_(NH) Flag-hGBP-1, the hGBP-1 cDNA was amplified from plasmid #516 (the gift of Peter Staeheli, University of Freiburg) and inserted into PCMV_2_(NH). Flag-tagged TUBB3 in pcDNA3.1 and pCMV *β*-gal were provided by Goufa Liu and Brian Ash-burner, repectively (University of Toledo).

### 2.2. Reagents

The following reagents were purchased from the indicated sources: rabbit anti-actin (A2066), Sigma-Aldrich; mouse monoclonal anti-*β*III tubulin (clone 5G8), Promega; mouse monoclonal anti-bromodeoxyuridine antibody (clone Bu2a), DakoCytomation; anti-phospho Histone H3, Cell Signaling; mouse anti-pan epithelial monoclonal antibody (MAB1631), rat monoclonal anti-hGBP-1 antibody (1B1; 1B1 antibody does not work on frozen sections), and mouse anti-human CD31 (CBL468) monoclonal antibody, Chemicon; rabbit polyclonal anti-human CD31 (ab28364) and mouse monoclonal (KP1) to CD68 (ab9555), Abcam; Recombinant human interferon gamma (hIFN-*γ*), PBL Biomedical Laboratories; paclitaxel, Calbiochem (cat# 580555).

### 2.3. Generation and Immunopurification of Polyclonal Anti-hGBP-1 Antisera

Rabbit polyclonal antisera against hGBP-1 were generated by New England Peptide, Inc using the sequence Ac-LKKGTSQKDETFNLC-amide for immunization. hGBP-1-specific immunoglobulins were isolated by immunopurification against hGBP-1 immobilized onto PVDF membranes.

### 2.4. SDS PAGE and Western Blot Analysis

Cells were lysed and proteins separated on SDS-PAGE, followed by transfer to PVDF membranes [12]. Membranes were probed with anti-hGBP-1 and anti-actin.

### 2.5. Patient Samples

Tumor samples were obtained from patients undergoing surgery for debulking of new or recurrent tumors. The patient cohort consists of White, Hispanic, and African- American women between the ages of 32 and 83. A table containing patient information is provided (Table 1). These studies were approved by the Institutional Review Board of the University of Toledo. All participants provided written informed consent. Tumor pieces were flash frozen in liquid nitrogen within 30 min of removal. Tissue samples of normal ovaries were provided by the Cooperative Human Tissue Network, funded by the National Cancer Institute.

### 2.6. RNA Isolation and Real Time RT-PCR

Total RNA was isolated from tissue samples using Qiagen RNeasy Kit (Qiagen Inc., Valencia, CA). For PCR of the tumor cDNAs, the TaqMan primers used were Hs00801390-sl for TUBB3, Hs00977005-ml for hGBP-1, and Hs03929097 for GAPDH per manufacturers instructions. All samples were assayed in triplicate in an ABI 7500 (Table 1).

### 2.7. Statistical Analysis

MANOVA was used to compare the patterns or levels of expression levels of the two genes from the normal, new, and recurrent groups. Wilk’s lambda one-way ANOVA was used to determine which genes were contributing to the global difference and which were not. Statistical tests were carried out on log (base 2) of the gene expression data because a log transformation was required to achieve normal distribution of values.

### 2.8. Immunofluorescence

Tumors sections (10 μm) were fixed in 4% paraformaldehyde. Sections were blocked for 2 hours followed by incubation with primary antibodies for 48 hours at 4°C: polyclonal hGBP-1 (1:50), anti-Mab1631 (1:3000), anti-CD31 (1:50), and anti-TUBB3 (1:1000). Slides were incubated with highly cross-adsorbed Alexa 594 conjugated anti-mouse (1:500) and Alexa 488-conjugated anti-rabbit (1:2000). Nuclei were stained with DRAQ5 (Cell Signaling Technology, Danvers, MA) (5 μM) or DAPI. Confocal images were collected using a TCS-SP spectrophotometric laser scanning confocal microscope (Leica Microsystems).

### 2.9. Tunel Assay

SKOV3 cells were plated onto coverslips in 6-well dishes and transfected with pCMV2-Flag or pCMV2 Flag-hGBP-1 and pCMV-*β*-gal at a 3:1 ratio. After 24 hours, the cells were treated with paclitaxel (5 μM) or vehicle (DMSO) for 18 hours, stained for *β*-gal, and analyzed for apoptosis by DeadEnd Fluorometric TUNEL system (Pro-mega Corp.).

### 2.10. Analysis of Mitotic Cells

SKOV3 cells were transfected as described. Cells were treated with 5μM paclitaxel or vehicle (DMSO) for 24 hours and analyzed for percent mitotic cells by indirect immunofluorescence for phosphohistone H3. At least 200 cells from each condition were scored as phosphohistone H3 positive or negative.

### 2.11. Progression-Free Survival

To determine progression-free survival (PFS) as a function of gene expression, KmPlot was used (http://kmplot.com/analysis/index.php?p=service&cancer=ovar). The 2015 version of the TCGA database with 1648 ovarian samples was screened for hGBP-1 expression with the Affymetrix probe of ID number 202269_x_at. The results are expressed as median progression-free survival for all stages, histologies, grades, and p53 status of ovarian cancers that underwent optimal debulking and chemotherapy that contained a taxol and platinum. For TUBB3 the search conditions were the same but the Affymetrix probe was 202154_x_at.

## 3. Results

### 3.1. hGBP-1 Protects SKOV3 Ovarian Cancer Cells from Paclitaxel-Induced Death in Vitro

**Figure 1:**
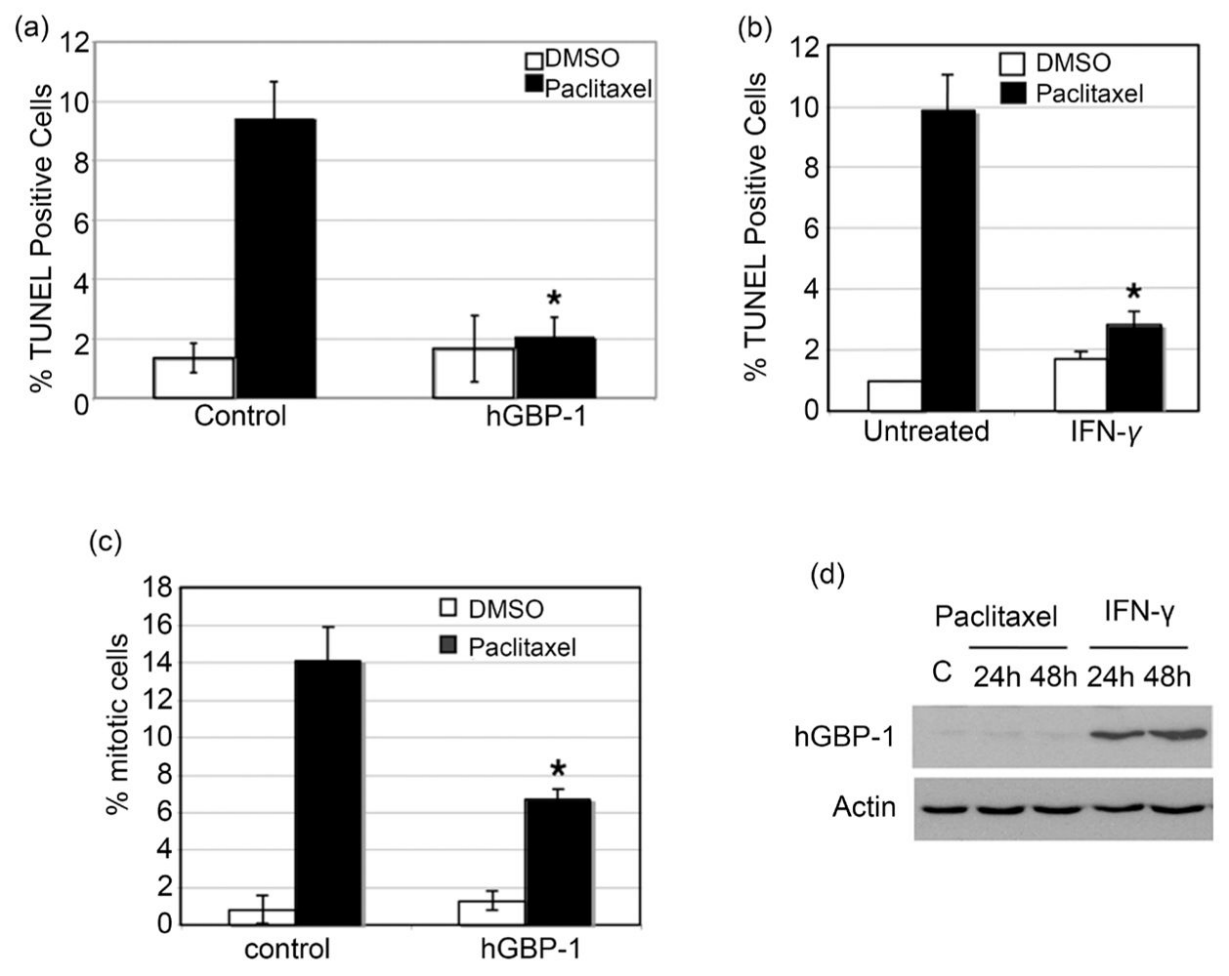
hGBP-1 blocks paclitaxel-induced death in SKOV3 cells. (a). Cells were transfected with Flag-hGBP-1 or control (empty vector) and pCMV-*β*-gal and treated with paclitaxel (5 μM) or vehicle (DMSO) for 18 hours, stained for *β*-gal expression, and analyzed by TUNEL. The results represent the average percent of *β*-gal positive cells that were also TUNEL positive ± SD (n = 3; * = p < 0.01). (b). SKOV3 cells were treated with IFN-*γ* (500 U/ml) or untreated and were examined by TUNEL assay after 24 hours. Results are expressed as mean TUNEL positive cells ± SD (n = 3; * = p < 0.05). (c). Cells were transfected with control vector or hGBP-1 and treated with 5 μM of paclitaxel or vehicle for 24 hours and stained for phosphohistone H3 and anti-FLAG. Results are expressed as mean percent mitotic cells ± SD (n = 3; * = p < 0.05). (d). Cells were plated for 24 hours and left untreated, or treated with paclitaxel (5 μM) or IFN-*γ* (500 U/ml) and analyzed for hGBP-1 and actin.

hGBP-1 makes ovarian cancer cells less sensitive to paclitaxel [3] [13]. To confirm that hGBP-1 protects ovarian cancer cells from paclitaxel-induced killing, SKOV3 cells lacking hGBP-1 (Figure 1(d)) were transfected with hGBP-1 and treated with paclitaxel. hGBP-1 blocked paclitaxel-induced apoptosis (Figure 1(a)). Induction of hGBP-1 in SKOV3 cells by IFN-*γ* also protects them from paclitaxel-induced apoptosis (Figure 1(b), Figure 1(d)). Paclitaxel kills cells, at least *in vitro*, by inducing mitotic block. Expression of hGBP-1 in SKOV3 cells reduces the number of paclitaxel-treated cells in mitosis (Figure 1(c)).

### 3.2. Paclitaxel Does Not Induce the Expression of hGBP-1 within 48 Hours

To determine whether paclitaxel-initiated intracellular signals directly induced the expression of hGBP-1, SKOV3 cells were treated with paclitaxel. Paclitaxel did not induce hGBP-1 expression within 48 hours (Figure 1(d)). These cells are competent to express hGBP-1, as evidenced by the induction of hGBP-1 by IFN-*γ*

### 3.3. Expression of hGBP-1 in Newly Diagnosed Ovarian Tumors

**Figure 2:**
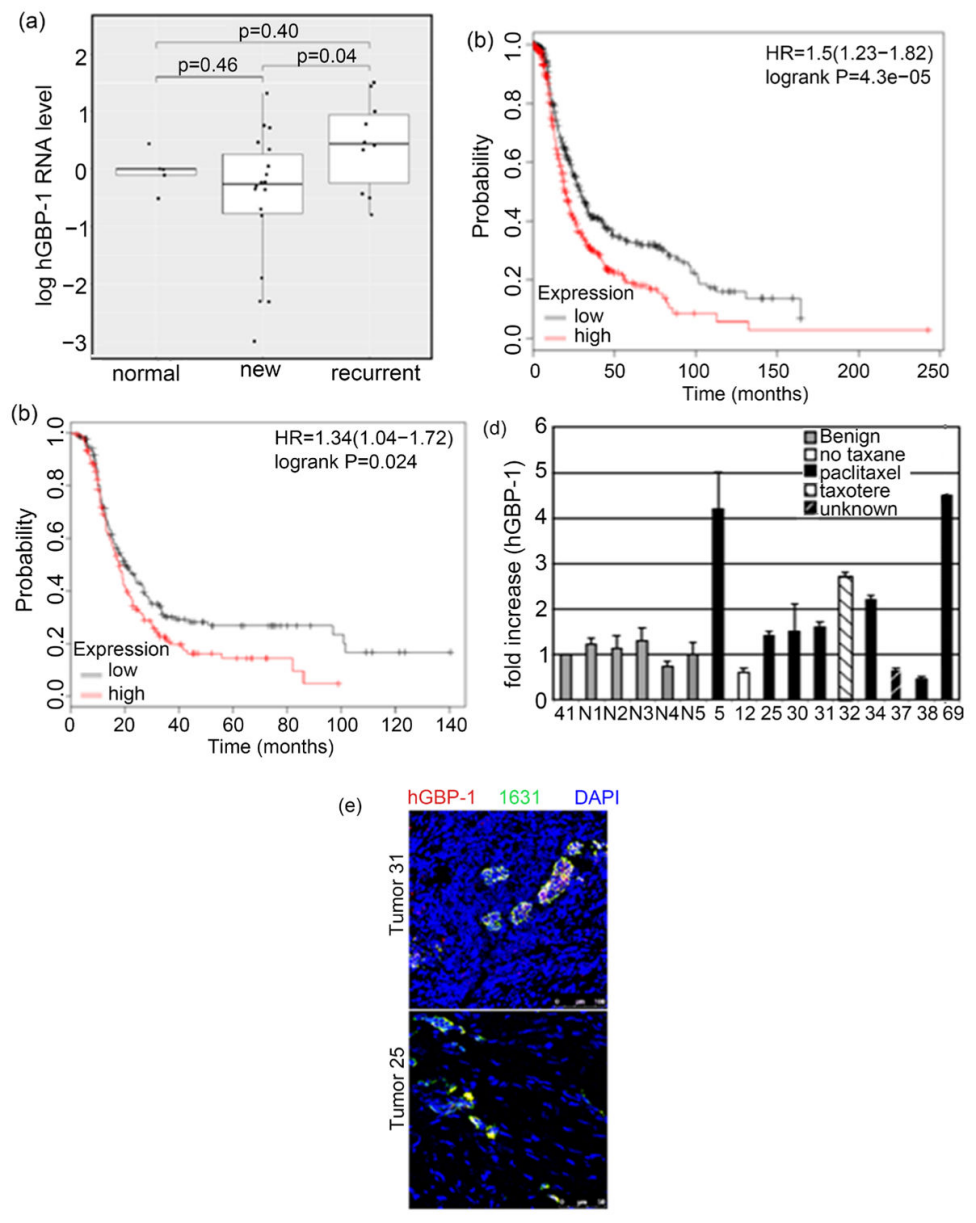
Expression of hGBP-1 mRNA in ovarian tumors. (a). Fold difference of hGBP-1 RNA in normal, new, and recurrent tumors was plotted for each individual tumor. (b). PFS was determined as described in Materials and Methods. The data from 340 tumors with low hGBP-1 and 341 tumors with high expression are shown. (c). PFS was determined from the tumors in part B but with the additional filter of subsequent treatment with paclitaxel and platin. (d). hGBP-1 RNA expression in recurrent ovarian cancers. (e). Sections from recurrent tumors were stained by indirect immunofluorescence for both Mab 1631 and hGBP-1.

All data on hGBP-1 and paclitaxel resistance were generated in cultured cells [2] [3] [13]. To examine the status of hGBP-1 expression in ovarian tumors, samples were obtained from women during surgery for initial diagnosis and debulking of ovarian cancer or from tumors that recurred after chemotherapy. Five normal ovary samples were obtained from The Comparative Human Tissue Network (CHTN) (Table 1). The mean value for hGBP-1 RNA in the benign ovarian sample 41 was set to 1 for comparison to tumor samples (Figure 2). The values of normal ovaries were close to the value for this sample. There was no significant difference between the levels of hGBP-1 mRNA in normal ovaries and those in newly identified ovarian tumors prior to chemotherapy. Of the 18 new tumors, only three (17% of the total 18) had hGBP-1 RNA levels 2-fold or greater compared to control (Figure 2(a)).

### 3.4. hGBP-1 Expression in New Ovarian Tumors Predicts Shorter PFS

To determine if the expression of hGBP-1 in newly isolated ovarian tumors altered disease prognosis, PFS of patients with ovarian tumors was determined for tumors with low or high initial hGBP-1 expression [10]. The data used for this analysis were limited to those tumors isolated from patients who underwent optimal debulking surgery. The data included ovarian tumors of all stages, histologies, grades, and p53 status. These filters reduced the 2015 TCGA database of 1648 ovarian tumors to 1306 patients. Matching tumors with high expression of hGBP-1 to those with low expression, further reduced the sample size to 341 tumors with high and 340 tumors with low expression. The PFS of patients with ovarian tumors with elevated levels of hGBP-1 is significantly shorter than that for tumors with low levels (Figure 2(b)). If the tumors are further filtered to include only those that were subsequently treated by chemotherapy that included a combination of paclitaxel and a platin, there were 174 with low levels of hGBP-1 and 175 with high levels of hGBP-1. These tumors with high hGBP-1 also have significantly shorter PFS (Figure 2(c)).

### 3.5. Expression of hGBP-1 in Recurrent Ovarian Cancers

The median level of hGBP-1 RNA in recurrent tumors was significantly higher than in new tumors (Figure 2(a)). The tumors were coded based on whether the patient had previously received a taxane as part of her chemotherapy (Figure 2(d)). Of the recurrent tumors, 7 of the 10 had hGBP-1 RNA values greater than controls, and 4 of the tumors had values greater than 2-fold higher than sample 41 (40%). However, laser capture micro-dissection was not used to separate tumor cells from surrounding cells. While the primary tumor samples were essentially 100% tumor, some of the recurrent samples contained lower percentages of tumor cells. In that case even if hGBP-1 expression was elevated within the tumor cells, the results for hGBP-1 RNA might not reach 2-fold elevation. To determine whether the recurrent tumors expressed hGBP-1, recurrent tumors number 5, 12, 25, 30, 32, 37, 38, and 69 were stained for both Mab 1631 and hGBP-1 (Figure 2(e)). All of these tumors expressed hGBP-1, except tumor 12. Interestingly this recurrent tumor was from a patient that had not received a taxane as part of her treatment regime. This raises the percentage of hGBP-1-positive recurrent tumors to at least 80%. It also reveals the flaws behind analyses of tumor samples, especially recurrent, that have not been laser capture microdissected.

### 3.6. Expression of TUBB3 in Ovarian Cancers *in Vivo*

**Figure 3:**
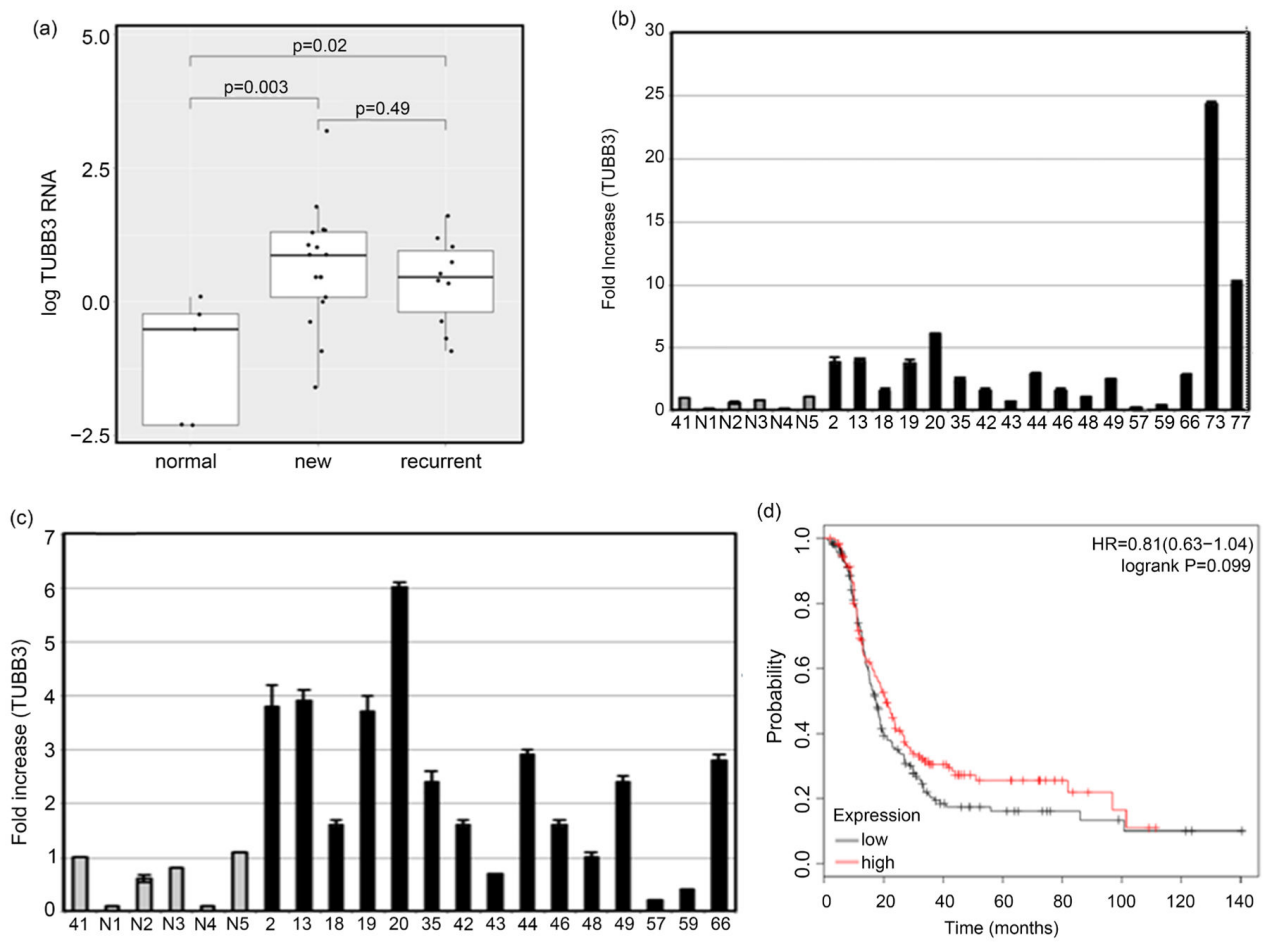
Expression of TUBB3 mRNA in ovarian cancers. (a). Fold increases of TUBB3 RNA in normal, new, and recurrent tumors. (b). Level of TUBB3 RNA in all new tumors of ovarian cancers. (c). New tumors are shown without tumors 73 and 77. (d). PFS was determined as described on 174 tumors with low TUBB3 and 175 tumors with high TUBB3.

TUBB3 has been associated with paclitaxel resistance *in vitro* and suggested to be regulated by hGBP-1 [13]. TUBB3 mRNA levels were higher in both new and recurrent tumors when compared to normal ovaries (Figure 3(a)). Of the 17 new tumors analyzed for TUBB3 RNA (Table 1), two had very high levels of expression (tumors 73 and 77) (Figure 3(b) and Figure 3(c)). TUBB3 RNA was up-regulated in 10 of 17 (59%) new ovarian tumors. When ovarian tumors of all histologies, grades, and stages were segregated into those with low and high TUBB3 expression, the differences in TUBB3 expression in ovarian cancers were not correlated with changes in PFS (Figure 3(d)).

### 3.7. Co-Expression of hGBP-1 and TUBB3 *in Vivo*

**Figure 4:**
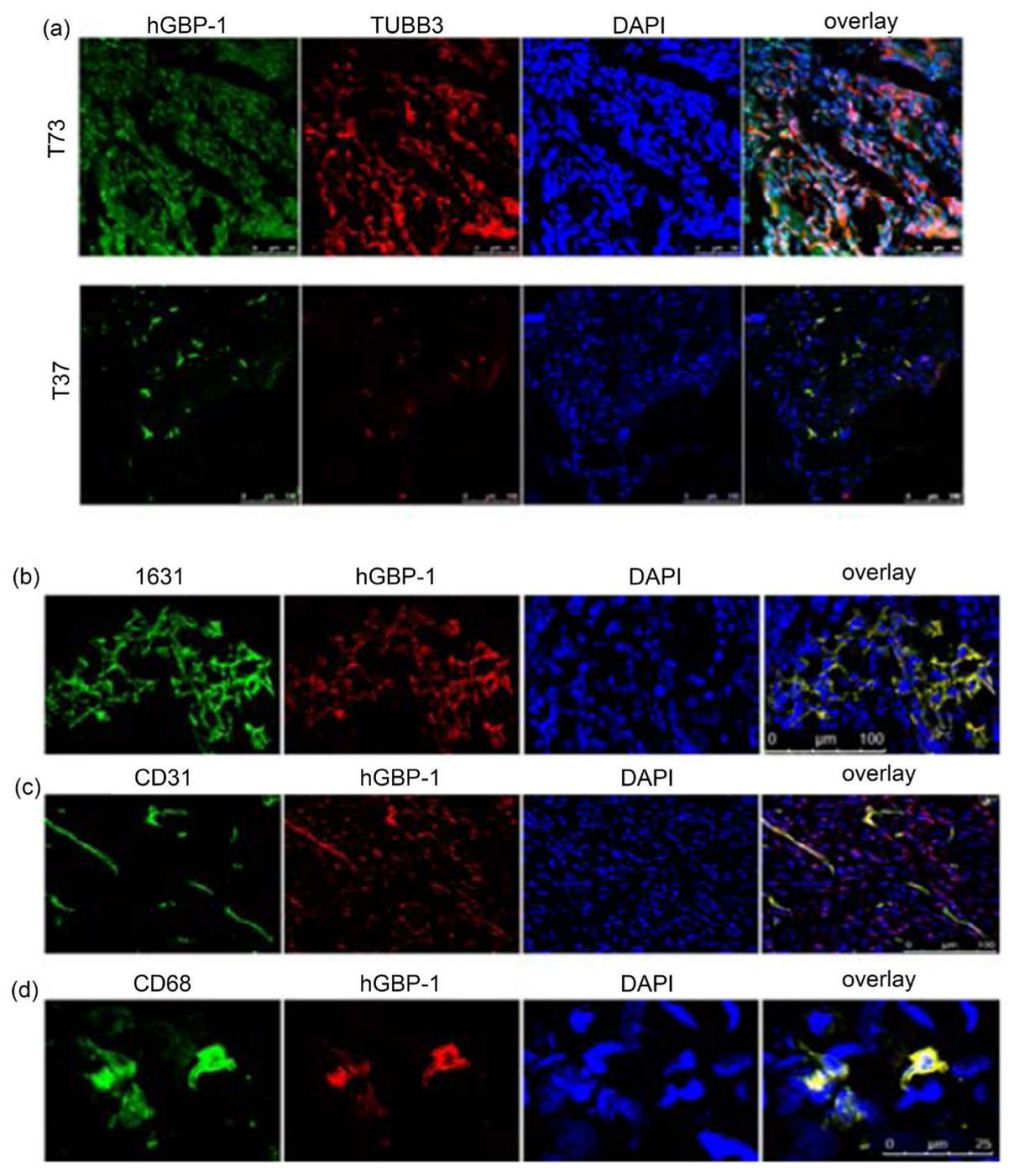
(a). Co-localization of hGBP-1 and TUBB3 in ovarian tumors. Frozen sections where examined for TUBB3 and hGBP-1 as described in Methods. The staining data from tumors 73 and 37 are shown. (b)-(d). Sections from tumor 13 were stained with affinity-purified anti- hGBP-1 antisera and Mab 1631 (b), anti-CD31 (c), or anti-CD68 (d).

To determine if hGBP-1 and TUBB3 are expressed in the same cells within the tumors, tumor sections were stained for hGBP-1 and TUBB3. In tumors such as tumor 73 with elevated expression of both hGBP-1 and TUBB3 (and it’s a primary tumor so the sample was all tumor) both proteins are expressed in all tumor cells (Figure 4(a)). In tumors such as tumor 37 where there were relatively few tumor cells within the sample, both proteins are co-expressed. However, not all recurrent tumors that expressed hGBP-1 also expressed TUBB3. Therefore, both hGBP-1 and TUBB3 may be expressed in the same tumor cells but it is not a prerequisite for the recurrent tumors.

### 3.8. Immunolocalization of hGBP-1 in Ovarian Tumors

Analysis of hGBP-1 mRNA levels from tissue samples does not identify the cell types expressing hGBP-1. In fact, IHC for hGBP-1 in breast cancers, where it correlates with improved prognosis, the protein is expressed strongly in tumors but also in the surrounding stroma [6]. To determine the cell types expressing hGBP-1 in ovarian cancers, flash frozen tumors were stained for hGBP-1 and co-stained for CD68 (macrophages), a pan-epithelial marker (Mab 1631), and CD31 (endothelial cells). Using an epithelial cell marker (Mab 1631), the tumor cells themselves robustly express hGBP-1 (Figure 4(b)). As previously demonstrated in a studyof colon cancers, hGBP -1 can also be expressed within endothelial cells (CD31) and monocytes (CD68) within the tumors (Figure 4(c) and Figure 4(d)). However, the hGBP-1-expressing cells within tumors are primarily tumor cells with few infiltrating hGBP-1-positive non-tumor cells. This is in contrast to observations of breast and colon cancer, where hGBP-1 is robustly expressed by infiltrating cells and surrounding stroma and is correlated with better prognosis [6][14] .

## 4. Discussion

Resistance to paclitaxel is a significant impediment to successful treatment of ovarian cancer. Understanding how ovarian tumor cells become resistant to paclitaxel would be an important first step toward developing ways to overcome this resistance. The large GTPase, hGBP-1, contributes to resistance to paclitaxel in cultured ovarian cancer cells [2] [3] [13] (Figure 1). What had yet to be examined was whether hGBP-1 was expressed in ovarian tumors and whether its pattern of expression correlated with paclitaxel resistance. While the current study contained a relatively limited number of patient samples, it still provided important information. hGBP-1 was expressed in only 17% of newly identified ovarian tumors that had not yet been treated (Figure 2). We also showed that elevated hGBP-1 in ovarian tumors is correlated with significantly shorter PFS (Figure 2). Although we only obtained 10 samples from women with recurrent tumors, 9 of the 10 were examined for hGBP-1 protein expression. The only tumor sample negative for hGBP-1 protein expression was the recurrent tumor from a patient who had not received either paclitaxel or docetaxel. Therefore, all of the tumor samples that we were able to examine and that came from patients with disease that returned after treatment with a taxane, were positive for hGBP-1. In this study, hGBP-1 was a more reliable marker for recurrence than TUBB3. In addition, hGBP-1 expression in recurrent tumors did not depend on the expression or activity of TUBB3.

All these data indicate that fewer than 20% of newly isolated ovarian tumors express elevated hGBP-1. Further, patients with those ovarian tumors initially expressing elevated hGBP-1 have a significantly shorter PFS than patients whose tumors do not. However, upon recurrence at least 80% of the tumors now express hGBP-1 strongly suggesting that those tumors that are initially negative or express hGBP-1 at low levels begin to express hGBP-1 as the tumors recur after therapies that include a taxane. Because ovarian cancer is not a very common cancer and the majority of patients with recurrent tumors do not have a secondary debulking surgery, the data for new and recurrent tumors from the same patient will be difficult to obtain. In fact, all published array data on gene expression changes in ovarian cancer accompanying drug resistance have been generated using ovarian cancer cells in culture or orthotopic growth of human tumors in mice. The array data that have been used to predict genes involved in drug resistance in ovarian cancer were obtained from tumors at initial debulking or biopsy and statistical analysis of PFS or survival was performed as a function of expression of particular genes.

Probing protein arrays of kinases with hGBP-1 identified an interaction with the kinase, PIM1 [13]. Molecular modeling also predicted hGBP-1 could interact with PIM1 [15]. Recently an inhibitor of this interaction was developed but its effect on sensitivity to paclitaxel has not been explored [16]. PIM1 has two isoforms [17]. This is the consequence of the use of two different translation start sites. The larger isoform is 44 kDa and the smaller is 33 kDa. Little is known about the functional differences between the isoforms. Most studies involve the 33 kDa isoform. Screening a panel of ovarian cancer cell lines that included ES2, OVCA194, OVCAR4, OVCAR3, and OVCA420 showed that all of the ovarian lines expressed the 33 kDa form of PIM1 [9], while the breast cancer cell lines, MCF-7, MCF-10A, MDA-MB-231 and MDA-MB-435 expressed the 44 kDa isoform of PIM1 [9]. The affymetrix probes used for generating the data in the TCGA database do not distinguish between the two isoforms. However, in ovarian cancer where the smaller isoform is expressed, PIM1 expression alone is not correlated with changes in RFS [9]. In breast cancer, where the larger isoform is expressed, elevated PIM1 correlated with improved RFS [9]. It is possible that the reason that hGBP-1 functions one way in ovarian cancer and another in breast cancer is because it interacts with adifferent isoform of PIM1.

This first examination of a role for hGBP-1 in paclitaxel resistance in ovarian patients provides evidence that is consistent with the cell culture data on cells that are paclitaxel resistant. In fact, it goes further. It shows that TUBB3 expression does not correlate with tumor recurrence after taxane treatment in ovarian cancer as well as hGBP-1 expression does.But it also leaves some questions unanswered. This pilot study should set the stage for additional studies on the role of hGBP-1 in treatment outcomes for ovarian patients.

## Figures and Tables

**Table 1:** Information for each tumor is: tumor number, patient age, race (C = Caucasian, H = Hispanic, AA = African American), diagnosis, tumor grade (if known), tumor stage (if known), whether the tumor was new (N) or recurrent (R), and the mean of the qRT-PCR results for hGBP-1 and TUBB3 (n = 2, in triplicate). There were no significant differences were between expression of hGBP-1 or TUBB3 in tumors based on age or race.

Tumor	Age	Race	Diagnosis	Grade	Stage	new/recurrent	GBP1	TUBB3
2	70	C	ovarian ca			N	1.05	3.8
5	79	C	ovarian ca		IIIC	R	4.2	5
12	67	C	ovarian ca			R	0.6	0.5
13	62	C	ovarian ca			N	3.75	3.9
18	73	C	ovarian adenocarcinoma		IIIC	N	0.8	1.6
19	57	C	ovarian papillary serous adenocarcinoma			N	1.6	3.7
20	70	C	ovarian ca			N	0.8	6
25	60	AA	ovarian adenocarcinoma		III	R	1.4	1.5
30	67	C	ovarian ca		III	R	1.5	2.1
31	64		ovarian ca	III	I	R	1.6	1.7
32	53	C	papillary serous adenocarcinoma			R	2.7	2.8
34	80	C	papillary serous adenocarcinoma		III/IV	R	2.2	0.7
35	83	C	ovarian ca	II	IIIB	N	0.7	2.4
37	78	C	ovarian adenocarcinoma		IIIC	R	0.65	3.3
38	60	C	ovarian adenocarcinoma		IIIC	R	0.45	1.4
41	49	C	cystic ovary				1	1
42	32	C	ovarian ca		IIIC/IV	N	0.5	1.6
43	59	C	ovarian ca		IIC	N	0.75	0.7
44	77	C	endometroid cancer of ovary		IC	N	0.05	2.9
46	72	C	primary peritoneal		IIIC	N	2.15	1.6
47	66	C	ovarian ca		IIIC	N	0.15	
48	51	C	ovarian ca		IV	N	0.9	1
49	68	H	transitional ovarian ca		IIC	N	1.4	2.4
57	69		papillary serous ovarian ca		IIIC	N	0.1	0.2
59	57	C	serous adenocarcinoma of ovary	III	IV	N	0.7	0.4
66	42	C	papillary serous adenocarcinoma of ovary		IIIC	N	0.45	2.8
69	70	C	papillary serous cystoadenocarcinoma of ovary	III	IIIA	R	4.5	0.4
73	65	C	papillary serous adenocarcinoma		IIIC	N	2.05	24.3
77	64	C	papillary serous adenocarcinoma		IIB	N	0.1	1.1
N1	63	C	normal ovary				1.55	0.1
N2	46	C	normal ovary				1	0.6
N3	51	C	normal ovary				1	0.8
N4	35	C	normal ovary				0.6	0.1
N5	66	C	normal ovary				0.9	1.1
